# Chickpea Proteins as Sustainable Ingredients: Techno-Functional Characterization

**DOI:** 10.3390/foods15061112

**Published:** 2026-03-23

**Authors:** Daniela Soto-Madrid, Sara Pérez, Camila Mella, Silvia Matiacevich, Rommy N. Zúñiga

**Affiliations:** 1 Department of Agro-Industry and Enology, Faculty of Agronomical Sciences, Universidad de Chile, Santa Rosa 11315, La Pintana, Santiago 8820808, Chile; 2 Department of Biotechnology, Universidad Tecnológica Metropolitana, Las Palmeras 3360, Ñuñoa, Santiago 7800003, Chilec.mellac@utem.cl (C.M.); 3 Department of Food Science and Technology, Technological Faculty, Universidad de Santiago de Chile, Av. Ecuador 3769, Estación Central, Santiago 9170022, Chile; silvia.matiacevich@usach.cl

**Keywords:** alternative proteins, physical properties, ingredients, plant-based

## Abstract

The growing consumer trend toward plant-based diets is prompting the food industry to seek alternatives to animal protein. Chickpea protein (CPP) stands out for its high protein content (14.9–24.6%) and represents a sustainable alternative. Therefore, this study evaluated and compared the techno-functional performance of CPP and whey protein isolate (WPI), with a focus on their emulsifying capabilities for plant-based food development. CPP was extracted via alkaline extraction and isoelectric precipitation. The techno-functional properties were evaluated, including solubility index (%), foaming capacity (%), emulsion activity index (EAI), gelling, and interfacial properties. Additionally, CPP was used as an emulsifier in plant-based emulsions, and the emulsion stability was compared with WPI for two months. Although CPP exhibited a lower solubility index (60 ± 1.0%) than WPI (95 ± 0.3%), its foaming capacity was identical (CPP: 57 ± 6%; WPI: 58 ± 4%) and exhibited a significantly higher emulsion activity index (22 ± 0.3 m^2^/g) than WPI (15 ± 0.8 m^2^/g). In terms of gelation, WPI formed stronger gels (1.2–2.1 N) than CPP (0.05–0.06 N), at the same concentrations. Interfacial tension measurements showed that, while CPP exhibited a higher interfacial saturation concentration (0.055 g/L vs. 0.023 g/L), it was more effective at reducing equilibrium interfacial tension than WPI. Finally, emulsion stability over two months was similar when using CPP or WPI as emulsifiers. CPP demonstrates a competitive functional profile; however, its implementation as a sustainable ingredient will require physical or chemical modifications to improve its functional properties for complex food matrices.

## 1. Introduction

Projections of a nearly 10-billion-person increase by 2050 raise critical questions about how to feed this growing population [1]. Thus, more nutritious foods are needed to ensure the health and well-being of future generations. In this context, the role of proteins becomes fundamental as they are essential macronutrients necessary for the growth, repair, and maintenance of body tissues [2]. Additionally, proteins are versatile ingredients in the food industry, employed by their functional properties and nutritional value. Proteins can act as structural building blocks in foods, influencing texture and water retention, and serve as thickeners, emulsifiers, foaming and gelling agents [2,3]. Common sources include animal proteins like milk, eggs, and meat, as well as plant-based options like soy, wheat, and legumes [4]. However, the increase in demand for proteins is limited by production capacity and available resources. Developing novel, protein-rich food alternatives is therefore crucial, balancing industrial innovation with the urgent need for environmental sustainability [5].

The consumption of animal protein has been an essential part of the diet of millions of people around the world for thousands of years, providing a source of essential nutrients. However, obtaining protein-rich foods from animals is not sustainable. The energy efficiency for meat production reaches values below 30%, and only 24% for milk production, which means that only 24% of the food ingested by the animal is converted into a product, while the rest is lost [6]. In addition to the low efficiency and high cost of animal protein production, the drawbacks associated with its excessive consumption are a warning sign, as the production of this type of food has consequences for human health and damage to the planet. Current industrial animal farming practices are widely regarded as unsustainable, producing 6.2 billion tons of CO_2_ equivalent (GtCO_2_eq) in 2015, contributing to 12% of total anthropogenic greenhouse gas emissions and about 40 percent of total emissions from agrifood systems. This figure is predicted to increase to 9.1 GtCO_2_eq by 2050; an increase of 46.8% [7]. Therefore, plant-based proteins have gained interest due to their sustainability and profitability compared to animal proteins. This supports the use of non-conventional protein sources such as plant proteins in the development of sustainable and functional foods [5,8].

Alternative proteins (e.g., plants, fungi, algae, and insects) represent an innovative option to the challenges of food security, human health, and environmental sustainability, offering a promising solution to ensure the availability of nutritious and accessible foods for the entire population, reducing the exploitation of natural resources, without compromising animal welfare [9]. Within this group, plant proteins are considered an excellent option for reducing the environmental footprint and represent a source of nutrients and functional ingredients of interest due to their diversity, availability, and cost-effectiveness. Vegetable proteins can be obtained mainly from legume seeds, cereals, oilseeds, and, to a lesser extent, green leaves [10,11,12,13]. Among these, soy and wheat stand out; however, the presence of allergens in the case of soy and the development of celiac disease associated with wheat consumption cause rejection by part of the population [11,12,14].

Chickpea (*Cicer arietinum* L.) is the third most abundant and second most widely consumed legume crop globally. Beyond its nutritional benefits, chickpea stands out as a highly sustainable protein source due to its natural drought resilience and low irrigation requirements [15]. Chickpea is characterized by its high protein content, typically 15–25% [16,17] These proteins are mainly globulins (~56%), glutelins (~18%), and albumins (~12%) [16,18]. The relative proportions of these fractions, along with their specific amino acid profiles, play a determinative role in the techno-functional behavior of chickpea protein. For instance, the high surface hydrophobicity of globulins and the interfacial stabilization capacity of glutelins are essential for the formation and maintenance of foams and emulsions [19]. This requires characterizing its structure, physicochemical, and techno-functional properties for the development of plant-based food ingredients.

Compared to other legumes, chickpea proteins have higher bioavailability, lower allergenicity, better solubility, and interfacial stabilization properties [17,19,20], making them a potential alternative that can replace animal proteins. Several studies have shown that chickpea protein can be used as an emulsifier to prepare and stabilize oil-in-water emulsions [17,19,21]. In addition, chickpea exhibits properties comparable to soy protein isolate, a widely used vegetable protein in meat products, including water retention, oil absorption, emulsification, and gelation [22]. Therefore, chickpea protein isolate (CPI) has excellent potential for application in food processing.

In the food industry, the development of innovative, high-quality products is driving the search for ingredients that, beyond their nutritional value, optimize the organoleptic and physical properties of the final product. In this context, plant proteins stand out as versatile ingredients due to their techno-functionality, which is intrinsically linked to a set of physicochemical attributes that govern their behavior during processing, storage, and consumption [5]. These critical parameters include composition, molecular structure, particle size, net charge, and molecular flexibility. According to Porras-Saavedra et al. [23], protein solubility does not strictly depend on overall amino acid composition but rather on the balance between hydrophilic and hydrophobic residues exposed at the surface. In this sense, surface hydrophobicity is a key determinant of a protein’s ability to interact with water and to establish its thermodynamic stability [5,24]. This amphiphilic nature allows proteins to migrate and adsorb at the oil–water interface, forming a protective film around oil droplets that prevents coalescence in emulsified systems [5,16]. The efficiency of this process is influenced by factors such as net charge and structural flexibility, which determine the protein’s capacity to reduce interfacial tension [19]. Indeed, a direct correlation exists between high solubility and emulsifying capacity, as rapid migration toward the interface is essential for the stability of complex systems such as dressings, dairy products, and processed meats [25]. Finally, the synergy between surface hydrophobicity and solubility also determines the gelation behavior of legume proteins, given that the unfolding of polypeptide chains is an indispensable prerequisite for the formation of stable, three-dimensional networks that define food texture.

The literature has reported that when comparing the techno-functional properties of three plant proteins—chickpea, soybean, and pea—chickpea protein stands out over the other legumes in terms of its water retention capacity, gelling property, and emulsifying capacity [26,27]. Water retention capacity is an important property to evaluate the use of plant-based ingredients in the production of food matrices similar to meat or yogurt [26]. On the other hand, the low concentration at which chickpea gels (5–7%) can be used to form semi-solid structures, such as cheese substitutes, and their emulsifying capacity allows them to be used in the manufacture of creams and bakery products [26,28]. These techno-functional properties of chickpea protein contribute to the choice of this food as a potential alternative source of protein for the replacement of animal proteins, such as whey protein, in the production of creams, yogurts, and cheeses, offering consumers a sustainable alternative. Although, despite the good properties of chickpea protein compared to other vegetable proteins, its use is not currently on a massive scale, as has happened in the case of soya protein, which has been widely used in food production.

In Chile, legume consumption—specifically chickpeas—experienced a steady decline for three decades leading up to 2018 [29]. However, this trend was reversed during the COVID-19 pandemic (2020), when consumption surged by 25% among the omnivorous population and 55% among vegetarians and vegans. This shift is attributed to chickpeas’ high protein content and their role as an accessible, low-cost component of a healthy diet [30]. Despite this renewed demand, domestic production has continued to dwindle due to low crop profitability and yields, as well as increased competition from Argentine and Canadian imports. Currently, chickpea cultivation is largely restricted to rainfed conditions (without irrigation). It is primarily maintained by small-scale farmers in the inland and coastal drylands of the O’Higgins and Maule regions, who dedicate their harvest to self-consumption and local markets [31,32].

Meanwhile, compared to soy protein, chickpea remains almost unexploited industrially in terms of its application for the formulation of foods with high nutritional quality and/or as a replacement for animal protein in the production of food products. Therefore, the objective of this study was to evaluate and compare the techno-functional properties of chickpea protein and whey protein isolate, with a specific focus on their emulsifying capabilities. Whey protein isolate (animal protein) was chosen because it is a widely used food ingredient derived from milk and has outstanding techno-functional properties. Thus, this study aims to provide new information about chickpea protein to support the sustainable development of plant-based food formulations.

## 2. Materials and Methods

### 2.1. Raw Materials

The chickpea protein (CPP) was extracted from commercial chickpea flour (Extrumol, Chile) according to the methodology of Soto-Madrid et al. [19]. The methodology is based on the solubility of the vegetable protein at alkaline pH (pH = 11.5) and precipitation at its isoelectric point (pH = 4.5). Subsequently, the protein was washed with purified water and neutralized at pH 7. Finally, the protein was freeze-dried (Virtis SP Scientific, Benchtop Pro 9LES-55, Warminster, PA, USA) and stored in a desiccator until further use. Protein content of the freeze-dried samples was 88.01 ± 6.07%. Commercial whey protein isolate (WPI) (BiPro, Agropur, Saint-Hubert, QC, Canada) was used as a control protein to compare its techno-functional and interfacial properties with CPP. All animal or vegetable protein dispersions were made with ultrapure water, hydrated at 4 °C overnight, then kept at room temperature for 1 h before being analyzed.

Flaxseed oil (Fontevita, Santiago, Chile), which is rich in polyunsaturated fatty acids, was used as the dispersed phase to evaluate interfacial properties and emulsion formulation.

### 2.2. Determination of the Techno-Functional Properties of Proteins

#### 2.2.1. Solubility Index

Solubility index determination was performed following the methodology of Arazo-Rusindo et al. [33], with slight modifications. Briefly, 2.5 g of protein was mixed with 25 mL of purified water, previously heated to 60 °C, and then shaken for 1 h on a magnetic stirrer at 500 rpm (DLAB, MS-H280-Pro, Beijing, China). Then, the sample was allowed to cool for 30 min and centrifuged at 4130 rpm (Restek, model Sep-3000, Bellefonte, PA, USA) for 20 min. At the end of this period, the supernatant obtained from the centrifugation was placed in a Petri dish and dried in a forced-air oven (Memmert, UF75, Schwabach, Germany) at 105 °C for 24 h. The solubility index was calculated using the following equation:

(1) $$Solubility\;index\left(\%\right)=\frac{supernatant\;dry\;weight\left(g\right)}{sample\;weight\left(g\right)}\times 100\%$$

#### 2.2.2. Foaming Capacity

The foaming capacity of proteins was evaluated by the method described by Lee et al. [34]. In this method, 100 mL of the protein dispersion (CPP or WPI) at 1% (*w*/*w*) was prepared and stirred at 10,000 rpm for 10 min in a rotor–stator homogenizer (Kinematica, PT2500e, Malters, Switzerland). Then, the sample was placed in a 250 mL graduated cylinder for 30 s to record the foam volume, and the foaming capacity was determined as a percentage (%), according to Equation (2).

(2) $$Foaming\;capacity\left(\%\right)=\frac{\left(Foam\;volume-Dispersion\;volume\right)}{Dispersion\;volume}\times 100\%$$

In addition, the foam’s half-life was measured, as the time (minutes) needed for the foam to lose 50% of its initial volume. Visual measurements were carried out on the same graduated cylinder to determine this time.

#### 2.2.3. Emulsion Activity Index (EAI)

The EAI of proteins was determined according to the method reported by Han et al. [35], with slight modifications. An O/W emulsion was prepared by dispersing flaxseed oil into the continuous phase, corresponding to the protein dispersion (CPP or WPI) at 1% (*w*/*w*). A dispersed phase of 30% (*w*/*w*) flaxseed oil was used to prepare the emulsion. The emulsion was fabricated using a rotor–stator homogenizer (Kinematica, PT2500e, Malters, Switzerland) at 10,000 rpm for 5 min. Immediately afterwards, the emulsion was diluted with a 0.1% (*w*/*v*) sodium dodecyl sulfate (SDS) solution until the absorbance at 500 nm was lower than 1.0. A 0.1% (*w*/*v*) SDS solution was used as the blank. The emulsion activity index (EAI) was calculated according to the following equation:

(3) $$EAI\left(m^{2}/g\right)=\frac{2\times 2.303\times A_{0}\times F}{C\times (1-\emptyset )\times 10^{4}}$$

where: A_0_ is the absorbance value at 0 min, Ø is the oil volume fraction, C is the protein concentration (g/mL), and F is the dilution factor of the emulsions, respectively.

### 2.3. Gelation Properties

#### 2.3.1. Dynamic Gel Formation

Gel formation was evaluated using an oscillatory test in a rheometer (Anton Paar, MCR 72, Graz, Austria) at a constant strain of 10%, with an angular frequency of 10 rad/s and a temperature of 80 °C for 45 min. Protein dispersions were analyzed at concentrations of 4, 5, 8, 10, 11, 12, and 13% (*w*/*w*). A rough parallel plate geometry with 50 mm in diameter (PP50 probe) was employed and set at 1 mm gap between plates. The evolution of the storage modulus (G′) was monitored to follow the gelation process; a sharp increase in G′ was considered the beginning of the gelation process. The following parameters were obtained from the G′ versus time graphs: (i) initial gelation time (min), corresponding to the initial point of the sharp increase in the slope; and (ii) final gelation time (min), corresponding to the final point of the slope.

#### 2.3.2. Minimum Gelation Concentration (MGC)

Food gels may be defined qualitatively through the tube inversion method, where a gel does not flow toward the direction of gravity when the container in which it was formed is inverted [10]. The minimum gelation concentration (MGC) of the proteins was evaluated according to the methodology described by Tang et al. [36]. Protein dispersions were prepared at 12, 13, 14, and 15% (*w*/*w*) for CPP and 13, 14, 15, and 16% (*w*/*w*) for WPI. Subsequently, the samples were placed in a temperature-controlled bath (Memmert, SV 45, Schwabach, Germany) at 80 °C for 30 min. After this time, the tubes were immersed in an ice bath for 30 min. The MGC was defined as the lowest protein concentration at which the sample did not slide down through the walls when the tubes were inverted and was expressed as a percentage (% *w*/*w*).

#### 2.3.3. Gel Strength

Protein dispersions (7.5 mL) at 15, 16, and 17% (*w*/*w*) for CPP and WPI were put in glass vials. Samples were heated at 80 °C for 1 h and cooled in ice water for 30 min to set the protein gels. Gel strength was tested in the same vials by a texture analysis machine (Zwick/Roell, model Z0.5, Zwick GmbH & Co., Ulm, Germany). A compression test was performed at a probe speed of 1 mm/s and 10 mm of penetration using a cylindrical probe of 10 mm in diameter. The gel strength was defined as the maximum force (N) measured during the test.

### 2.4. Determination of Interfacial Properties of Proteins

#### 2.4.1. Dynamic Interfacial Tension Measurements

The dynamic interfacial tension of the proteins was determined by the pendant drop method [19]. In this method, an axisymmetric drop of protein dispersion was delivered and allowed to stand at the tip of a steel needle inside a quartz cell containing 30 mL of flaxseed oil at 25 °C, to achieve protein adsorption at the oil–water interface. An optical tensiometer (Ramé-Hart, model 250-F4, Succasunna, NJ, USA) was used to measure changes in interfacial tension (mN/m) over 90 min. This instrument uses a camera to detect changes in droplet shape as the protein is adsorbed at the interface. The DROPimage Advanced software v.3.1808 (Ramé-Hart Inc., Succasunna, NJ, USA) fits a polynomial equation to the droplet shape to calculate the interfacial tension.

Protein dispersions at different concentrations (0.005–0.5 g/L for CPP and 0.005–0.05 g/L for WPI) were used as the aqueous phase, and flaxseed oil as the lipid phase. Before measurements, the oil was purified with resin (Florisil^®^ 60–100 mesh, 46385, Sigma Aldrich, Darmstadt, Germany) at a resin:oil ratio of 1:10 to remove surfactant impurities. The mixture was stirred for 4 h and then centrifuged (Hettich, 320 R, Hemelingen, Germany) at 3500 rpm for 30 min. The oil was then filtered through a 0.45 µm syringe filter (Finetech, Taichung, Taiwan) and stored at 5 °C until use.

#### 2.4.2. Interfacial Saturation Concentration (ISC)

Interfacial saturation concentration (ISC) is defined as the concentration of protein in dispersion at which no more protein can be adsorbed due to saturation of the oil–water interface. At this concentration, no further decrease in interfacial tension can be produced. Results for interfacial tension at equilibrium (90 min) as a function of the concentration of the protein dispersions used in point 2.4.1 were plotted, and a linear regression analysis was performed, obtaining two linear relationships. First, interfacial tension decreased as protein concentration increased, and then, it remained unaffected by further increases in protein concentration. The point at which both linear relationships intersected was defined as the ISC.

### 2.5. Physical Stability of Plant-Based Emulsion

#### 2.5.1. Fabrication of O/W Emulsion Using Chickpea Protein as Emulsifier

O/W emulsions (200 g) were prepared by dispersing flaxseed oil (dispersed phase) in the continuous phase as described in Section 2.2.3. For these emulsions, the continuous phase was made of 3% or 6% (*w*/*w*) CPP dispersion. The emulsions were subjected to a second stage of high-pressure homogenization (Panda Plus 2000, GEA, Parma, Italy) at 500 bar for 1 cycle. WPI dispersions (3% and 6% *w*/*w*) were used as controls to evaluate the physical stability of O/W emulsions.

#### 2.5.2. Physical Stability of Emulsions

All emulsion samples were stored in glass test tubes (6.5 cm high) with lids under refrigerated conditions (5 °C) for 63 days. The stability of the emulsions was determined using static light diffraction on a vertical scanning analyzer (Formulaction, Turbiscan MA2000, Toulouse, France). The backscattering of an incident near-infrared light (λ = 880 nm) was measured automatically every 40 μm along the sample height.

The Turbiscan Stability Index (TSI) was used to evaluate emulsion stability because it is a statistical parameter that estimates dispersion stability, which considers the sum of all the destabilization processes of a dispersion, allowing us to compare stability over time. This parameter was calculated according to Equation (4) [37]:

(4) $$\mathrm{TSI}\;=\;\Sigma_{n}\left(\frac{\Sigma_{h}|{scan}_{i}\left(h\right)-{scan}_{i-1}\left(h\right)|}{H}\right)$$

where scan_i_ (h) represents the backscatter at a certain height for a specific measurement time (i) (%), scan_i−1_(h) represents the backscatter at the same height for the previous measurement time (i − 1) (%), and H is the sample height (mm). This comprehensive approach provides a detailed assessment of emulsion stability under controlled conditions. Higher TSI values indicate higher instability, whereas lower TSI values indicate more stable emulsions, as evidenced by minimal variations in backscattered light compared to initial conditions. These variations imply a reduced occurrence of phenomena such as coalescence, flocculation, sedimentation, and creaming [37,38].

#### 2.5.3. Microstructure and Visual Analysis of Emulsions

The emulsion microstructure was characterized qualitatively using an optical microscope (Olympus, model CKX53, Tokyo, Japan) equipped with a 40× objective. A small volume of the emulsion (5 µL) was placed on a microscope slide and covered with a coverslip. The micrographs were acquired using the microscope image analysis software (LCmicro 2.2, Olympus Soft Imaging Solutions GmbH, Olympus, Tokyo, Japan) during storage. In addition, images of the samples in the Turbiscan test tubes were captured with a digital camera (DSC RX100 V, Sony, Tokyo, Japan) during the storage time.

### 2.6. Statistical Analysis

All statistical analyses were performed in triplicate using three independent samples. Data were reported as means with their corresponding standard deviation. ANOVA test at a 95% confidence level was used to determine statistical differences using Statgraphics Centurion XVIII^®^ software v.18.1.12 (StatPoint Technologies Inc., Warrenton, VA, USA). Differences between samples were evaluated using the Least Significant Differences (LSD) multiple comparison method.

## 3. Results and Discussion

### 3.1. Techno-Functional Properties of Proteins

The incorporation of protein concentrates and isolates enhances nutritional value and offers specific functionalities to food products. However, plant proteins present differences from animal proteins in primary, secondary, and tertiary structures, as plant and animal proteins play different biological roles, which have an impact on their techno-functional properties. Some techno-functional properties of chickpea protein (CPP) are shown in Table 1, compared with whey protein isolate (WPI), a common animal protein used in the food industry.

Protein solubility plays a critical role in determining the functionality of proteins in food products. It relates to the balance of protein–solvent interactions, affecting functional properties such as emulsification and foaming [26]. Knowledge of protein solubility has a major impact on its utilization as a functional ingredient for food development [8]. It is reported that the solubility of plant protein ranges from 8% to 80%, depending on intrinsic factors, such as the plant source, amino acid profile, protein structure, and extrinsic factors like processing method, pH, ionic strength, and temperature [11,13]. However, the functionality of plant proteins is often impaired by their low water solubility [10].

This study evaluated and compared the solubility of CPP to the solubility of WPI. According to Table 1, the solubility of CPP is 60 ± 1.0%, a value considerably lower (*p* < 0.05) than 95 ± 0.3% for WPI. The elevated solubility of WPI can be attributed to the compact globular nature of its protein fractions, which exhibit high levels of solubility across a broad pH range [39]. The low solubility of CPP may be related to the extraction method used and protein structure. The extraction methodology, which is based on solubilization at an alkaline pH followed by precipitation at the isoelectric point, favors protein aggregation and globulin precipitation, which are the most abundant protein fractions in chickpeas [40,41]. The high surface hydrophobicity of globulins negatively affects protein–solvent interactions, thereby impairing the solubility of CPP [19] The solubility of proteins has important consequences for food formulation and processing. The low solubility and poor dispersibility of CPP are especially relevant for the formulation of low viscosity foods and beverages where proteins are prone to sedimentation, such as protein-rich drinks [8,13].

Regarding foam formation, no statistical differences were observed between the two proteins (*p* > 0.05), with a foaming capacity of 57.3% ± 6.3% for CPP and 57.7% ± 4.1% for WPI (Table 1). A similar pattern was observed for the foam half-life values (Table 1), with no significant differences (*p* > 0.05). Although plant proteins have shown limited foaming capacity due to their compact structures, which slow their adsorption at the air–water interface [11], CPP has an identical foaming capacity to WPI. For plant proteins, globulin fractions generally form foams with poor stability because of their aggregated structure, which forms a weak interfacial film. In contrast, the albumin fraction shows foam stability comparable to whey proteins due to cohesive interfacial layer formation around air bubbles [10]. Despite WPI having a higher solubility index than CPP, our results indicate that the foaming capacity and foam half-life of CPP and WPI were identical. This is because surface hydrophobicity and the availability of free sulfhydryl groups in proteins influence foaming properties, enabling proteins to adsorb and reorganize at the air–water interface, thereby rapidly encapsulating air bubbles [11,40]. CPP has shown higher values of surface hydrophobicity than animal proteins, which explains the results obtained [19].

Finally, the emulsion activity index (EAI), which reflects a protein’s ability to stabilize oil-in-water emulsions [42], was evaluated. This parameter depends on solubility, conformational flexibility, and the exposure of polar and hydrophobic groups to the interface [11,43]. Table 1 shows that the EAI is higher for CPP (22 ± 0.3 m^2^/g) than for WPI (15 ± 0.8 m^2^/g). This is consistent with results reported for CPP concentrates (~25 m^2^/g) obtained by isoelectric precipitation [44]. The literature also indicates that CPP concentrate exhibits higher EAI values than other vegetable protein concentrates, such as those from peas and lentils [45,46]. This evidence supports the potential of CPP to stabilize food emulsions, demonstrating that, despite differences in solubility, CPP has foaming and emulsification properties comparable to those of WPI. These findings position CPP as a valuable functional ingredient, opening new possibilities for developing more sustainable and nutritious plant-based foods.

### 3.2. Gelation Properties

#### 3.2.1. Dynamic Gel Formation

Protein gelation refers to the transition from a sol state to a solid-like structure, which plays a crucial role in structuring food systems [11]. Heat treatment is one of the most common methods used for food protein gelation, and the ability of proteins to form gels that retain water, flavors, and other ingredients is crucial for developing new food products and improving food functionality [47]. For this reason, the present study conducted an oscillatory assay to evaluate the gelation time of CPP and WPI dispersions.

**Figure 1:**
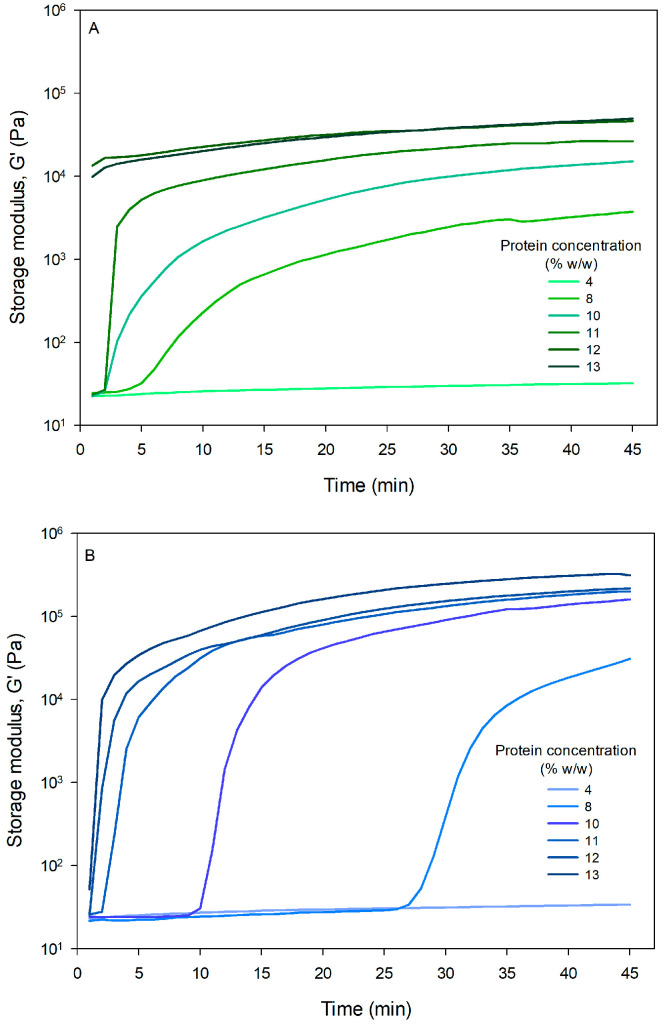
Dynamic gel formation. (**A**) chickpea protein and (**B**) whey protein isolate.

The results indicate that CPP forms a semi-solid structure in a significantly shorter time than WPI (Figure 1). For instance, at a concentration of 8% (*w*/*w*), the initial gelation time was 5.0 ± 1.0 min for CPP and 27.7 ± 0.6 min for WPI (Table 2). The lower gelation times of CPP are due to its composition and denaturation temperature. The extraction method used in this work extracts globulins, which are primarily responsible for CPP gelation properties [40]. The difference in gelation times between the two proteins is related to their denaturation temperatures. According to Kornet et al. [48], the liquid/gel transition for WPI occurs at about 80 °C, the denaturation temperature of its main component, β-lactoglobulin. In contrast, chickpea globulins undergo this transition at a lower temperature of around 60 °C, which explains why gel formation requires less time, as the rheometer was set at 80 °C for the oscillatory experiments. Furthermore, the gelation time for both proteins decreased with increasing concentration. This behavior could be attributed to increased protein–protein interactions at higher concentrations, which facilitate the formation of a gel network more quickly.

The storage modulus (G′) measures the elastic energy stored in the gel and reflects its stiffness or firmness. WPI gels had a significantly higher G′ value than CPP gels (Table 2) due to the presence of covalent (disulfide bonds) and non-covalent (electrostatic and hydrophobic) interactions in whey proteins [36], which are essential to form a uniform, firm gel network. In plant protein dispersions, high temperatures are used to aggregate and agglomerate protein molecules by exposing hydrophobic sites. Above the gelation onset temperature, proteins begin forming aggregates, which would subsequently form a continuous network of aggregated protein agglomerates [10]. The greater stiffness of the WPI network is evident in Figure 1.

The results of this analysis demonstrate that CPP can be used to formulate foods with a semi-solid or gel structure, as they can form a gel network at low concentrations. It is known that gel formation is strongly determined by the surface conformations of proteins (e.g., free sulfuryl groups, hydrophobicity, surface charge) and interaction between proteins (covalent bonds, hydrogen bonds, hydrophobic interactions, electrostatic interactions) [36]. Plant proteins can form physical gels, which are a product of noncovalent interactions between molecules that are induced by heat [10]. However, in the case of CPP, it was demonstrated that a neutral pH disulfide covalent bonds between protein subunits (legumins) were formed, but it may not be the major force involved in gel formation for these proteins [36]. The kind of bonds in CPP gels (mostly noncovalent) can explain why they are significantly weaker than WPI gels, as evidenced by their lower storage modulus (G′). At neutral pH, WPI gels are formed by covalent bonds between β-lactoglobulin molecules, forming a fine strand type of protein gel with higher strength than plant protein gels [49]. This suggests that CPP is ideal for applications that do not require high rigidity, offering promising potential for developing gelled products with soft textures.

#### 3.2.2. Minimum Gelation Concentration (MGC)

**Figure 2:**
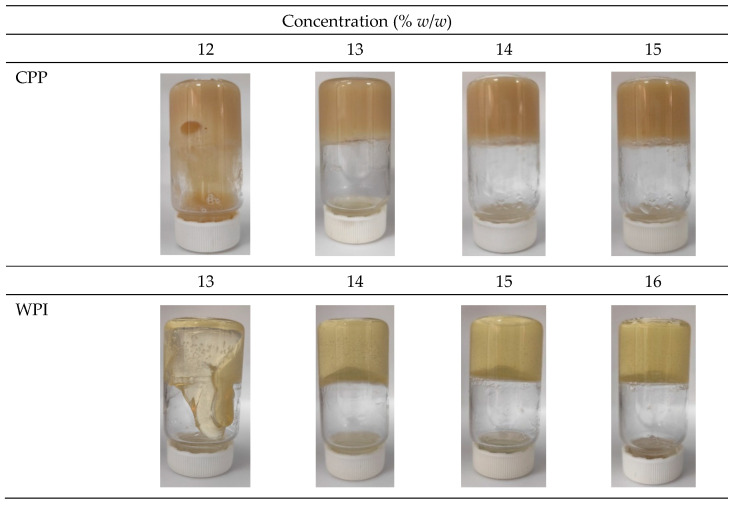
Photographs of samples used to determine the minimum gelation concentration (MGC) for chickpea protein (CPP) and whey protein isolate (WPI).

The minimum gel concentration (MGC) was compared for both protein gels. CPP exhibits a lower MGC (13% *w*/*w*) compared to WPI, which requires a concentration of 14% (*w*/*w*) to form a gel (Figure 2). This result is consistent with the findings of the dynamic gelation analysis. Values of MGC between 5 and 7% were reported for CPP concentrate with a protein content in the range of 71 to 77%, and an MGC of 12% was obtained for CPP isolate with a protein content of 89% [26]. As stated before, differences in techno-functional properties could be related to cultivar and extraction method, among other factors, as well as gelation conditions, such as pH, ionic strength, and heating conditions.

As shown in Figure 2, the gels also differ visually in terms of color and brightness. WPI gels are more translucent due to the formation of soluble protein complexes. At neutral pH, the formation of aggregates enables the creation of an ordered, translucent gel network. In contrast, CPP gels are noticeably opaque. This visual difference is due to the proteins having a lower surface charge density, which promotes the formation of disordered cross-linked strands that scatter lighter and affect the gel’s transparency [36].

#### 3.2.3. Gel Strength

The strength of the CPP and WPI gels was evaluated using a compression test to determine their resistance to deformation [50]. Gels were prepared at three concentrations (15%, 16%, and 17% *w*/*w*), and their gel strength was compared at the same concentrations (Table 3).

WPI gels exhibited significantly higher gel strengths at all three tested concentrations, ranging from 24 to 35 times those of CPP (Table 3). These results demonstrate that, although CPP can form gels, they are substantially weaker than those formed by the animal protein WPI. It is consistent with previous findings, such as those reported by Moussaoui et al. [51], who compared legume gels made from chickpea, lentil, and red lentil flours. They noted that CPP gels tend to be softer and less rubbery, resulting in lower shear strength. Despite this low firmness, Moussaoui et al. [51] suggest that the ability of CPP to form soft gels makes it suitable for producing solid, sliceable products such as sausages, thereby highlighting its functional potential in specific food applications.

### 3.3. Determination of Interfacial Properties of Proteins

#### 3.3.1. Dynamic Interfacial Tension Measurements

Amphiphilic molecules, such as proteins, play a critical role in stabilizing emulsions by adsorbing at the oil–water interface. This process not only reduces the system’s free energy, facilitating droplet breakup during homogenization, but also allows the formation of an interfacial film that provides physical stability through repulsive and steric interactions [52,53]. Hence, understanding the absorption behavior and interfacial properties of proteins at the oil/water interface is essential for stable emulsion production.

**Figure 3:**
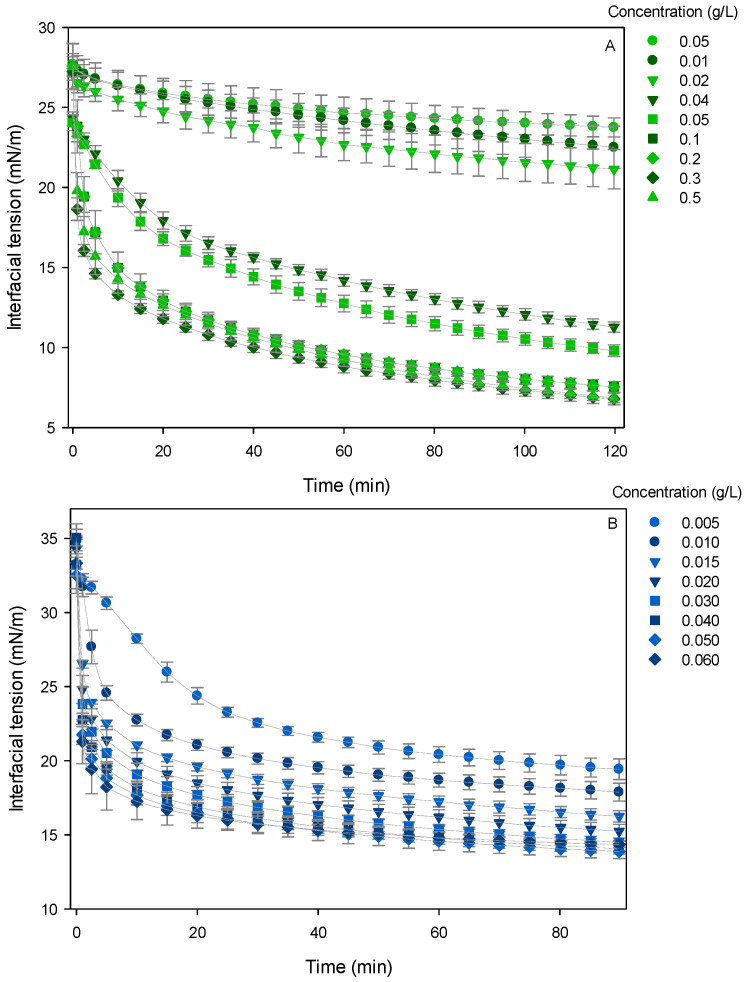
Dynamic interfacial tension of plant and animal proteins at different concentrations. (**A**) chickpea protein and (**B**) whey protein isolate.

In this context, the dynamic interfacial tension of CPP was compared with WPI using the pendant drop method. Figure 3 shows the different kinetic behaviors of the two proteins: while WPI exhibited rapid diffusion to the interface, CPP showed slower adsorption kinetics, characteristic of the higher molecular weight of plant proteins or their complex quaternary aggregates. Nevertheless, CPP reduced interfacial tension to significantly lower values (~7 mN/m) than WPI (~14 mN/m). These results suggest that, despite the slower diffusion towards the interface, the plant protein is more effective at unfolding and reorienting its hydrophobic residues towards the oil phase. This drastic reduction in interfacial tension not only facilitates droplet breakup during homogenization but also promotes the formation of a viscoelastic interfacial film that protects against coalescence. As noted by Yang et al. [54], this reduction in interfacial tension is a critical determinant of overall emulsifying efficiency.

#### 3.3.2. Interfacial Saturation Concentration (ISC)

Interfacial saturation concentration (ISC) is defined as the minimum surfactant concentration required to fully cover the oil–water interface, at which point interfacial tension reaches a stable minimum value and becomes concentration-independent [37,55].

**Figure 4:**
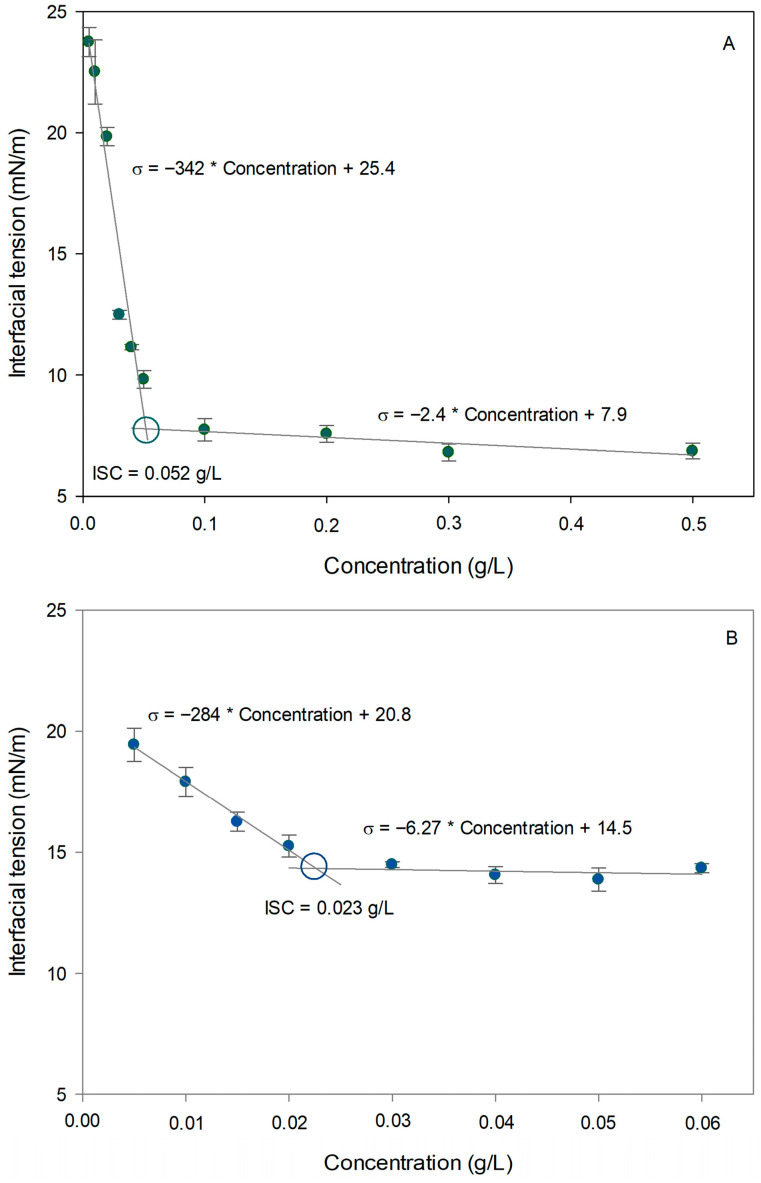
Interfacial saturation concentration (ISC) for (**A**) chickpea protein and (**B**) whey protein isolate. Circles represent the intersection in both lines, corresponding to the ISC.

Figure 4 illustrates the determination of ISC for CPP and WPI at the flaxseed oil interface across different concentrations. Both proteins exhibited characteristic surfactant behavior, with interfacial tension decreasing progressively with increasing biopolymer concentration, indicating their adsorption at the interface. The estimated ISC values were determined from the intersection of the linear regions corresponding to the tension-reduction phase and the saturation plateau [37].

The ISC value for CPP was higher (0.052 g/L) than WPI (0.023 g/L). It indicates that, although a higher mass of plant protein is required to saturate the interface, likely due to its higher molecular weight or aggregate size, it can reduce interfacial tension to significantly lower values once saturation is achieved. The results obtained highlight the potential of CPP as a highly effective stabilizer for plant-based emulsion formulations, in line with the results obtained by Soto-Madrid et al. [19], when comparing CPP with commercial ovalbumin. In addition, it was stated that among the functional properties of plant proteins, the ability to form and stabilize emulsions stands out as a critical application in food products [13].

The techno-functional performance of CPP is intrinsically linked to its compositional profile, particularly the balance between its main protein fractions (albumin, globulin, and glutelin) and their specific amino acid composition. In the study by Soto-Madrid et al. [19], the high emulsifying and foaming capacities reported are supported by the significant presence of hydrophobic amino acids (25.4 g/100 g in total extraction). These residues, including leucine, phenylalanine, and isoleucine, are crucial for adsorbing the protein at air/water and oil/water interfaces, effectively reducing interfacial tension. Furthermore, the compositional analysis indicates that while globulins (11S and 7S) provide superior surface hydrophobicity—reaching indices up to 5.7 times higher than animal proteins like ovalbumin—the glutelin fraction contributes significantly to surface pressure (34 mN/m), which is key for foam stabilization. This synergy between fractions, governed by an electrostatic profile that ensures stability at neutral pH (Zeta potential < −30 mV), explains the robust functional performance of CPP as a sustainable food ingredient [19].

### 3.4. Application of CPP in Plant-Based Emulsion

#### 3.4.1. Physical Stability of Emulsions

**Figure 5:**
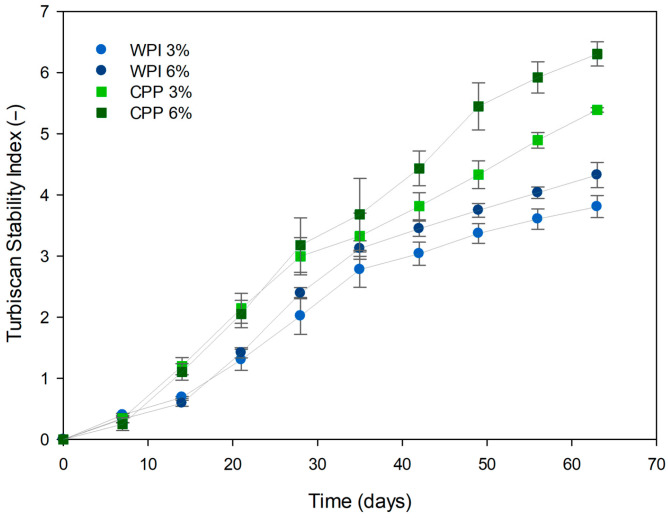
Turbiscan Stability Index (TSI) for oil-in-water emulsions stabilized with chickpea protein and whey protein isolate for 63 days.

The stability of the O/W emulsions at 3% and 6% (*w*/*w*) of protein was evaluated over 63 days using the Turbiscan Stability Index (TSI). All formulations showed an upward trend in TSI values over time (Figure 5), indicating progressive destabilization driven by phenomena such as coalescence, flocculation or creaming [37,38].

Emulsions stabilized with 6% (*w*/*w*) CPP showed the greatest instability of all samples, reaching a final TSI value of ~6.3. This result indicates that the high concentration (6% *w*/*w*) of CPP, which is typically less soluble and more globular/aggregated [42], exceeds the critical flocculation concentration. This excess protein promotes flocculation, in which the protein saturates the interface but leaves segments exposed that can bind to multiple droplets, resulting in rapid deterioration of stability as detected by the TSI.

In contrast, formulations stabilized with WPI maintained superior long-term stability. It should be noted that the 3% (*w*/*w*) WPI emulsion was the most stable throughout the study (final TSI ~3.8). This finding is crucial, as it suggests that 3% (*w*/*w*) was the optimal concentration for WPI, allowing the formation of an efficient interfacial layer without promoting the instability that could have occurred with 6% (*w*/*w*) WPI (final TSI ~ 4.3).

Although the 3% (*w*/*w*) CPP emulsions exhibited comparable initial performance to the WPI controls, the rate of destabilization (slope of the curve) accelerated notably starting from day 15. However, the TSI at 3% (*w*/*w*) for both proteins was relatively close on day 35. However, the difference was amplified considerably by the end of the storage. Upon concluding the experiment (day 63), 3% (*w*/*w*) CPP (TSI ~5.4) was markedly less stable than both WPI formulations.

It is important to note that the variations detected by the Turbiscan with TSI values below 3 correspond to the onset of destabilization; however, destabilizations remain non-visual in most cases (>90%) [56] and were observed for up to 28 days for CPPs (Figure 5). Consequently, CPP was able to stabilize emulsions, particularly at concentrations of 3% (*w*/*w*), where enhanced adsorption and coverage of the oil-water interface occur. However, long-term stability was inferior to that of WPI, suggesting that CPP, as a sustainable functional ingredient, requires physical or chemical modifications to optimize its resistance to destabilization mechanisms. Physical modification by thermal and non-thermal methods has been proposed to improve solubilization and emulsification properties of plant proteins, as well as chemical and enzyme treatments [13].

#### 3.4.2. Microstructure and Visual Analysis of Emulsion

**Figure 6:**
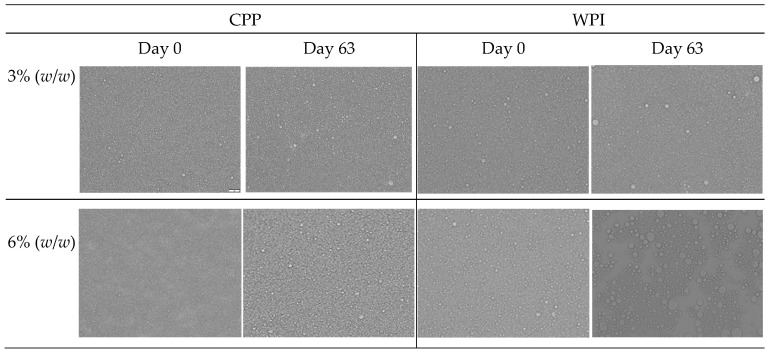
Optical micrographs of O/W emulsions stabilized by chickpea protein and whey protein isolate stored at 5 °C for 63 days. The bar corresponds to 20 µm.

Microscopy images of the emulsions show no visual differences between samples fabricated at 3% or 6% (*w*/*w*) of CPP, with droplet diameters smaller than 10 μm (Figure 6). In addition, no visual differences can be seen from images taken over the 63 days of storage, with a slight increase in diameters (additional information in the Supplementary Material). These results indicate that the destabilization phenomenon quantified by TSI was not due to coalescence, confirming the capacity of CCP to generate an interfacial layer that maintains the integrity of individual droplets. In contrast, WPI stabilized emulsions at 3% (*w*/*w*) showed a slight increase in diameters at day 63, and coalescence was evident for WPI stabilized emulsions at 6% (*w*/*w*) at the end of the storage.

**Figure 7:**
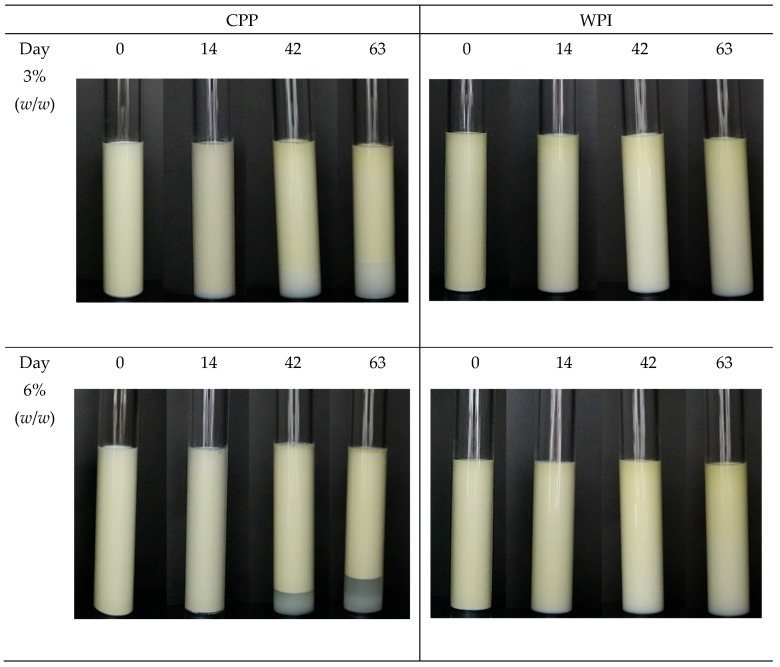
Photographs of the physical stability of O/W emulsions stabilized with 3% (*w*/*w*) and 6% (*w*/*w*) chickpea protein (CPP) and whey protein isolated (WPI) stored at 5 °C for 63 days.

Visual analysis of the CPP stabilized emulsions showed destabilization by creaming, the lower part of the tubes was depleted of oil droplets, and clarification was observed as storage time increased (Figure 7). Emulsions formulated at 3% (*w*/*w*) of CPP started to cream on day 14; whereas emulsions formulated at 6% (*w*/*w*) started to cream at day 35, two weeks later (additional information in the Supplementary Material). However, emulsions at 6% (*w*/*w*) of CPP showed a clearer serum layer at the bottom of the tubes. Probably, the higher concentration of CPP in these emulsions provoked a higher interaction between proteins at the interfacial layer of droplets, accelerating the gravitational movement of droplets, thus increasing the destabilization of these emulsions during storage time, as quantified by TSI values. Therefore, from these analyses, we can conclude that destabilization of CPP-stabilized emulsions was mainly due to creaming, which was protein concentration dependent.

In the case of WPI stabilized emulsion, only a slight clarification was observed at the bottom of the tube for both protein concentrations, confirming the higher stability quantified by means of TSI values, and indicating that destabilization was mainly due to coalescence.

## 4. Conclusions

This study provided comparative information on the techno-functional properties and stability of food emulsions, using plant (chickpea protein, CPP) and animal (whey protein isolate, WPI) protein.

Despite its significantly lower solubility (60%) compared to WPI (95%), CPP showed foam formation and stability comparable to WPI. This behavior suggests that the higher surface hydrophobicity of CPP compensates for its lower solubility, enabling efficient adsorption at the air–water interface. In terms of emulsifying capacity, CPP outperformed WPI, with respective emulsifying activity index (EAI) scores of 22 m^2^/g and 15 m^2^/g. Interfacial characterization revealed that, although CPP has slower adsorption kinetics and requires a higher concentration to saturate the interface (0.055 g/L compared to 0.023 g/L for WPI), it reduces interfacial tension to significantly lower values (CPP = ~7 mN/m; WPI = ~14 mN/m). This results in greater coverage and protection of oil droplets, establishing CPP as a highly effective stabilizing agent for sustainable formulations. Concerning gel properties, CPP was more effective at forming structural networks, with a lower minimum gelling concentration of 13% (*w*/*w*) compared to 14% (*w*/*w*) for WPI. However, CPP gels were significantly less rigid (with a lower elastic modulus, G′) than WPI gels, making them ideal for products requiring soft or semi-solid textures. Conversely, the destabilization of CPP-stabilized O/W emulsions was primarily attributed to creaming, dependent on the protein concentration used.

Taken together, these findings confirm that CPP is a valuable and sustainable functional ingredient, and it can effectively replace animal proteins across various food applications, providing valuable information for the development of high-quality plant-based products.

## Figures and Tables

**Table 1:** Techno-functional properties of chickpea protein and whey protein isolate.

Sample	Solubility Index (%)	Foaming Capacity (%)	Foam Half Life (min)	Emulsion Activity Index (m^2^/g)
CPP	60 ± 1.0 ^a^	57 ± 6 ^a^	4.7 ± 0.3 ^a^	22 ± 0.3 ^b^
WPI	95 ± 0.3 ^b^	58 ± 4 ^a^	4.3 ± 0.4 ^a^	15 ± 0.8 ^a^

Different letters (a,b) indicate significant differences (*p* < 0.05) between samples.

**Table 2:** Gelation times and elastic modulus (G′) for the dynamic formation of chickpea protein and whey protein isolate gels.

Concentration (% *w*/*w*)	CPP			WPI		
	Initial Gelation Time (min)	Final Gelation Time (min)	Final G′ Value (Pa)	Initial Gelation Time (min)	Final Gelation Time (min)	Final G′ Value (Pa)
8	5.0 ± 1.0 ^c^	16.7 ± 2.1 ^d^	3748 ± 2008 ^a^	27.7 ± 0.6 ^d^	33.3 ± 0.6 ^d^	30,734 ± 1962 ^a^
10	3.7 ± 2.0 ^c^	11.0 ± 3.0 ^c^	15,070 ± 7245 ^ab^	11.3 ± 1.2 ^c^	14.7 ± 1.5 ^c^	190,175 ± 34,005 ^b^
11	2.0 ± 0.0 ^b^	4.0 ± 1.0 ^b^	26,360 ± 19,780 ^b^	3.0 ± 2.0 ^b^	6.0 ± 1.7 ^b^	289,715 ± 3825 ^c^
12	0.0 ^a^	0.0 ^a^	46,243 ± 6922 ^c^	1.7 ± 0.6 ^ab^	3.7 ± 0.6 ^a^	318,270 ± 30,165 ^c^
13	0.0 ^a^	0.0 ^a^	49,341 ± 2092 ^c^	1.0 ± 0.0 ^a^	2.7 ± 0.6 ^a^	316,280 ± 82,152 ^c^

Different letters (a,b,c,d) indicate significant differences (*p* < 0.05) between samples.

**Table 3:** Gel strength for chickpea protein (CPP) and whey protein isolate (WPI) gels.

Sample	Concentration (% *w*/*w*)	Gel Strength (N)
CPP	15	0.05 ± 0.010 ^a^
	16	0.06 ± 0.004 ^ab^
	17	0.06 ± 0.001 ^b^
WPI	15	1.22 ± 0.02 ^c^
	16	1.99 ± 0.11 ^d^
	17	2.09 ± 0.17 ^d^

Different letters (a,b,c,d) indicate significant differences (*p* < 0.05) between samples.

## Data Availability

The original contributions presented in this study are included in the article/Supplementary Material. Further inquiries can be directed to the corresponding authors.

## Supplementary Materials

The following supporting information can be downloaded at: https://www.mdpi.com/article/10.3390/foods15061112/s1, Figure S1. Photographs of the physical stability of O/W emulsions stabilized with 3% (*w*/*w*) and 6% (*w*/*w*) chickpea protein stored at 5 °C for 63 days; Figure S2. Photographs of the physical stability of O/W emulsions stabilized with 3% (*w*/*w*) and 6% (*w*/*w*) whey protein isolate stored at 5 °C for 63 days; Figure S3. Optical micrographs of O/W emulsions stabilized by chickpea protein (CPP). The bar corresponds to 20 mm; Figure S4. Optical micrographs of O/W emulsions stabilized by whey protein isolate (WPI). The bar corresponds to 20 mm.
