# Supplementary material for: Chickpea Proteins as Sustainable Ingredients: Techno-Functional Characterization
Source: Foods. 2026 Mar 23;15(6):1112. doi: 10.3390/foods15061112 (PMC13025650; doi:10.3390/foods15061112)
Supplement: Supplementary file 1 [file foods-15-01112-s001.zip › foods-4127556-supplementary.pdf]

Supplementary Material

CPP 3% (w/w)

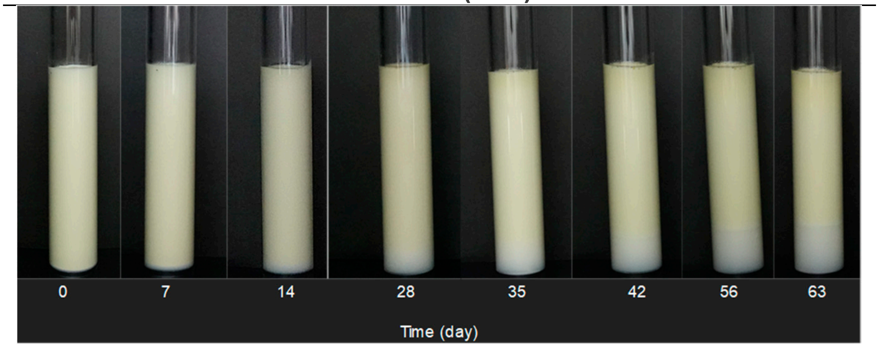

CPP 6% (w/w)

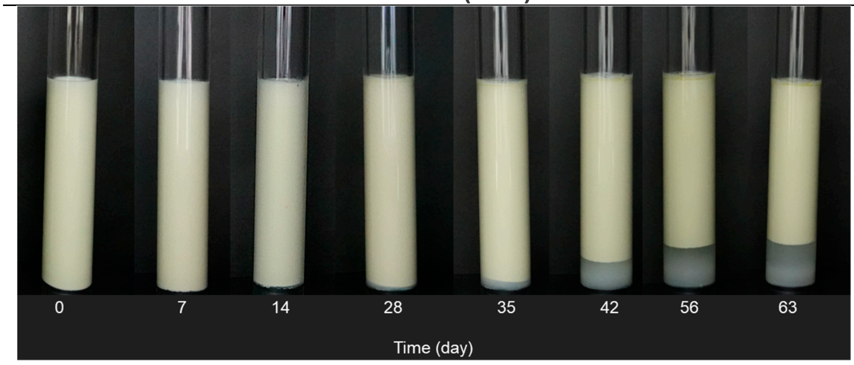

Figure S1. Photographs of the physical stability of O/W emulsions stabilized with 3% (w/w) and 6% (w/w) chickpea protein stored at 5 °C for 63 days.

WPI 3% (w/w)

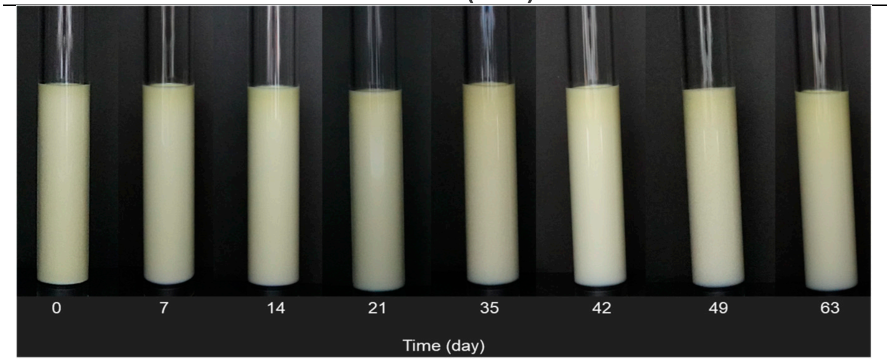

WPI 6% (w/w)

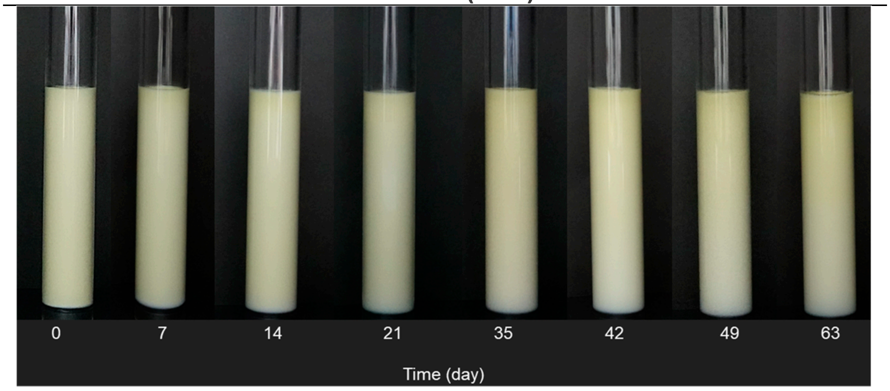

Figure S2. Photographs of the physical stability of O/W emulsions stabilized with 3% (w/w) and 6% (w/w) whey protein isolate stored at 5 °C for 63 days.

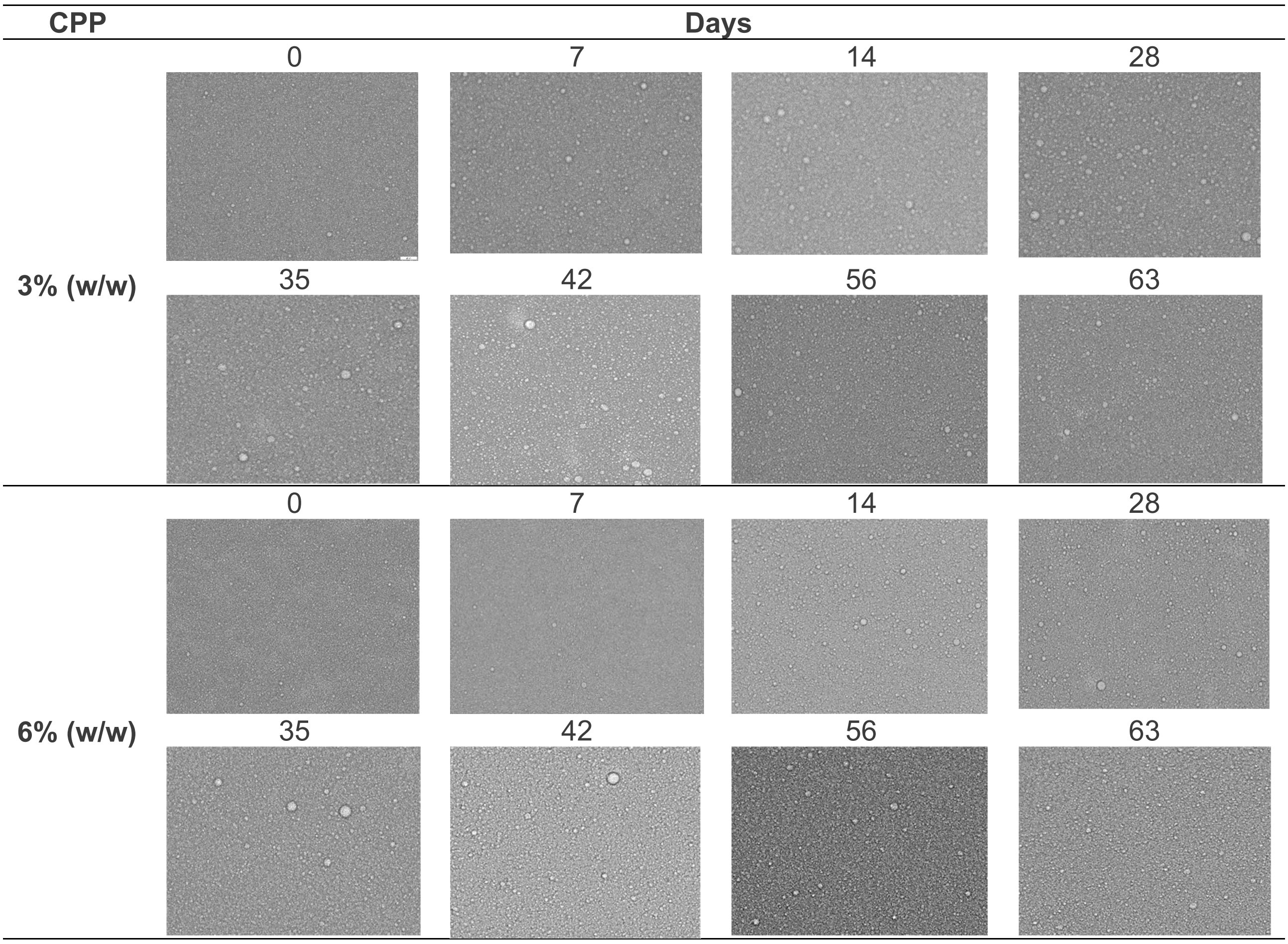

Figure S3. Optical micrographs of O/W emulsions stabilized by chickpea protein (CPP). The bar corresponds to 20 μm.

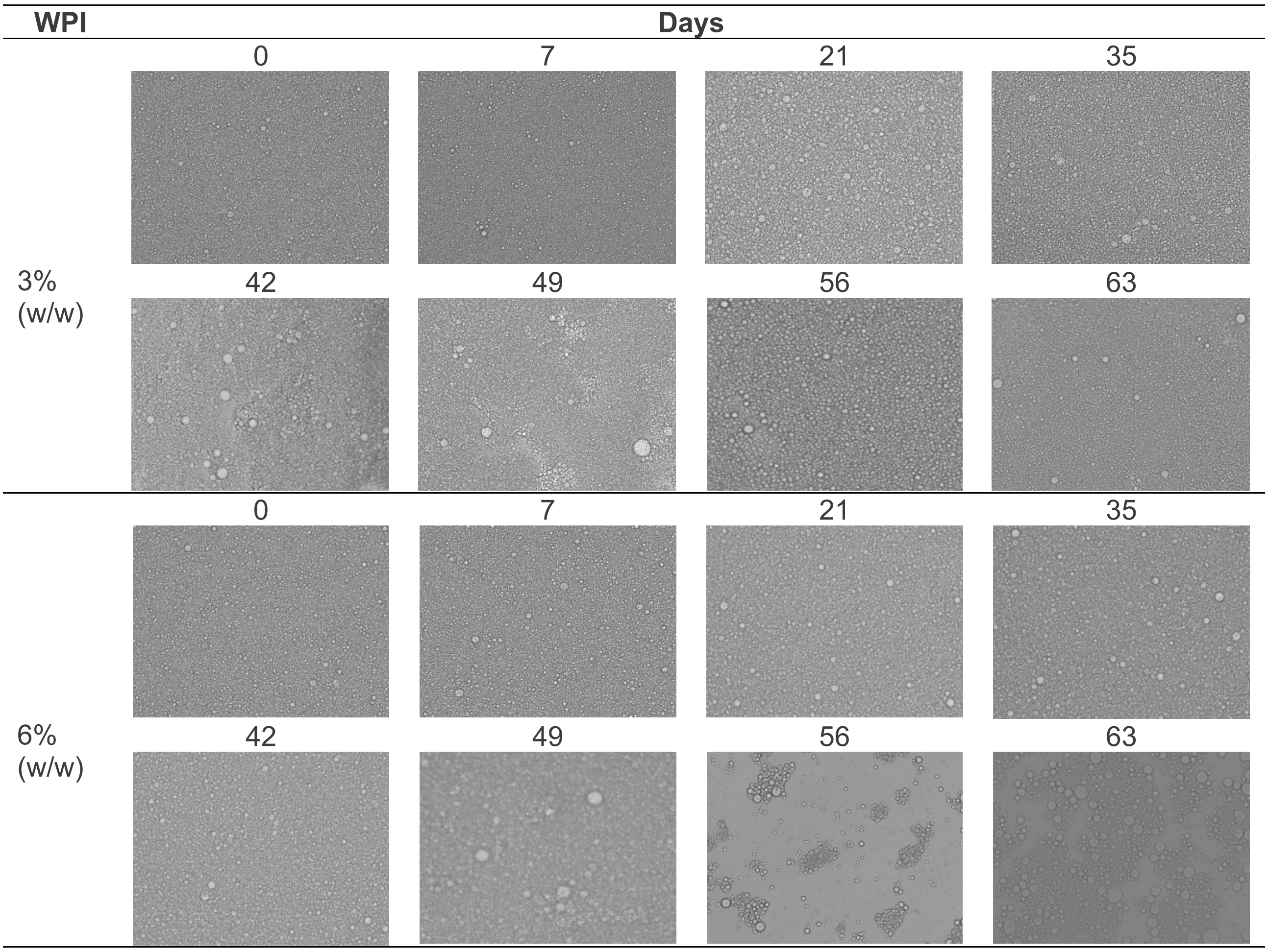

Figure S4. Optical micrographs of O/W emulsions stabilized by whey protein isolate (WPI). The bar corresponds to 20 μm.
